# EKV-VBQ: Ensuring Verifiable Boolean Queries in Encrypted Key-Value Stores

**DOI:** 10.3390/s24216792

**Published:** 2024-10-22

**Authors:** Yuxi Li, Jingjing Chen, Fucai Zhou, Dong Ji

**Affiliations:** 1 School of Computer Science and Engineering, Northeastern University, Shenyang 110819, China; 2 Software College, Northeastern University, Shenyang 110819, China; 3 National Frontiers Science Center for Industrial Intelligence and Systems Optimization, Northeastern University, Shenyang 110819, China

**Keywords:** verifiable Boolean queries, encrypted key-value stores, blockchain, homomorphic encryption, bilinear accumulators

## Abstract

To address the deficiencies in privacy-preserving expressive query and verification mechanisms in outsourced key-value stores, we propose EKV-VBQ, a scheme designed to ensure verifiable Boolean queries over encrypted key-value data. We have integrated blockchain and homomorphic Xor operations and pseudo-random functions to create a secure and verifiable datastore, while enabling efficient encrypted Boolean queries. Additionally, we have designed a lightweight verification protocol using bilinear map accumulators to guarantee the correctness of Boolean query results. Our security analysis demonstrates that EKV-VBQ is secure against adaptive chosen label attacks (*IND-CLA*) and guarantees *Integrity and Unforgeability* under the bilinear *q*-strong Diffie–Hellman assumption. Our performance evaluations showed reduced server-side storage overhead, efficient proof generation, and a significant reduction in user-side computational complexity by a factor of log *n*. Finally, GPU-accelerated optimizations significantly enhance EKV-VBQ’s performance, reducing computational overhead by up to 50%, making EKV-VBQ highly efficient and suitable for deployment in environments with limited computational resources.

## 1. Introduction

The proliferation of microservices architecture, real-time data processing, and big data analytics has significantly increased the demand for efficient and scalable key-value storage solutions. Vast streams of key-value data, such as sensing records and time-series data, collected and aggregated from numerous terminals, possess considerable value for statistical analysis. Such analysis is crucial for supporting research and decision making, which, in turn, generates substantial demands for data storage and computational resources. Many popular technologies and platforms, including Amazon DynamoDB [1], Redis [2], Memcached [3], and Cassandra [4], rely on key-value storage as their fundamental data model. These platforms are widely adopted across industries, including e-commerce, finance, social media, and IoT. While users depend on these platforms for analyzing, processing, and sharing key-value data, they do not guarantee data security and user privacy. Therefore, designing secure and private key-value stores is a critical challenge, especially as these outsourced platforms increasingly store sensitive data.

To ensure the confidentiality of outsourced key-value data and prevent unauthorized access by cloud service providers and hackers, data owners typically encrypt their data before outsourcing it to the cloud. However, conventional encryption schemes struggle to effectively match both keys and their corresponding values, which must be encrypted to prevent attackers from inferring the actual content through patterns in the encrypted data. Additionally, the latency introduced by encryption computation can impede real-time data analysis, which is crucial for key-value streaming data. Thus, traditional encryption methods face significant challenges in meeting the performance and real-time requirements of key-value data analysis. Encrypted search [5,6,7] has been proposed, to address this issue, allowing cloud servers to search encrypted data without decrypting it. These technologies ensure the confidentiality and usability of outsourced data, enabling users to safely and efficiently utilize outsourced data storage services. However, many existing encrypted search systems fall short in the following areas:

Inadequate support for Boolean queries: Boolean operations, such as conjunction (AND), disjunction (OR), and negation (NOT), are frequently used in key-value queries, to retrieve specific data records based on multiple conditions. For key-value stores, the values can be highly flexible (such as JSON, BLOB, or other complex objects). Boolean queries enable more granular filtering and selection based on subfields or specific attributes within the values. This flexibility makes key-value data stores applicable not only to simple key-value lookups but also to more complex data access requirements. Existing encrypted search techniques often lack support for expressive Boolean queries. Some approaches that do support Boolean queries [8,9,10] suffer from significant computational burdens, especially on the client side, which is often resource-constrained. This limitation reduces their usability, as real-world applications typically require a combination of various conditions in their queries.

Inefficiency in correctness verification: Boolean queries increase the likelihood of incorrect results due to node crashes or external attacks. Therefore, it is imperative to develop a verification mechanism to ensure the correctness of encrypted Boolean query results. Some existing approaches employ data authentication techniques, such as MAC authentication structures [11], Merkle hash trees [12], and Bloom filters [13], to verify query results. Another solution is to store key-value data in blockchain as transactions. In the proposed schemes [14,15,16,17], the consensus characteristic of blockchain guarantees that users can obtain reliable and correct search results containing the search keywords without needing additional verification, as long as the transactions are correctly run on blockchains. The consensus mechanism ensures that each node holds a consistent copy of the data and verifies the correctness and immutability of query results through multi-node validation.

However, these methods either offer limited expressiveness in validating query requests or suffer from heavy computational loads, making them unsuitable for verifying Boolean encrypted key-value queries. Therefore, improving query expressiveness while reducing the user verification cost is a pressing issue that must be addressed in verifiable encrypted searches.

Our contributions: This paper presents EKV-VBQ, a novel scheme designed to ensure verifiable Boolean queries in encrypted key-value datastores. EKV-VBQ integrates blockchain technology as part of the key-value datastore, guaranteeing query integrity and efficient secure appends. The scheme introduces a novel verification mechanism that ensures Boolean query results are computed correctly without tampering or forgery—improving upon previous approaches. Additionally, by leveraging GPU-optimized proof generation, EKV-VBQ significantly reduces computational overhead, making it suitable for resource-constrained mobile devices. Our key contributions are as follows:

- Formalization of EKV-VBQ: We formalize EKV-VBQ by defining its syntax through four polynomial-time algorithms/protocols: EKV-VBQ = (Init, Append, Query, Verify). We abstract the information leaked during these procedures, identifying how it can be used by an adversary. Furthermore, we provide formal definitions of semantic security against adaptive chosen label attacks (IND-CLA), Integrity and Unforgeability, outlining the specific security goals that EKV-VBQ achieves.
- Blockchain-integrated collaborative Boolean query: EKV-VBQ integrates a distributed, blockchain-secured key-value datastore that guarantees efficient secure appends without decryption. A chained key-value storage structure, indexed by keys, is stored on the blockchain, while the server only maintains a lookup dictionary indexing the blockchain addresses of each key. The server collaborates with the blockchain to perform efficient encrypted Boolean key-value queries. The immutability and decentralized trust mechanism provided by Ethereum’s proof of stake (PoS) consensus ensure that query integrity is preserved, even in the presence of malicious actors. This approach design avoids the high storage and update overheads typically associated with constructing auxiliary index structures.
- Efficient verification mechanism with bilinear accumulators: To ensure both the integrity and correctness of Boolean query results, we propose a lightweight verification protocol for EKV-VBQ based on bilinear map accumulators and the extended Euclidean algorithm. This mechanism allows users to verify that query results have not been tampered with, even in the event of a compromised service provider. We also formally define and rigorously prove EKV-VBQ’s Unforgeability, based on the bilinear *q*-strong Diffie–Hellman assumption.
- GPU-accelerated optimized implementation: We implemented a prototype of EKV-VBQ and evaluated its performance using Google LevelDB. Our experimental results demonstrate that the server-side proof generation time in EKV-VBQ consistently remains around 2.5 s, with verification optimization accounting for only 22% to 26% of the server-side computation. We also implemented GPU-accelerated optimizations, to further enhance the efficiency of EKV-VBQ. By parallelizing the proof generation process and leveraging GPU computing, EKV-VBQ achieved significant performance improvements in both query processing and proof generation, reducing computational overhead by up to 50%. These optimizations make EKV-VBQ highly practical for deployment in environments with limited computational resources.

## 2. Related Works

### 2.1. Verifiable Encrypted Search

The concept of verifiable encrypted search was first introduced and explored by Chai et al. in 2013 [18]. In 2015, Wang et al. proposed an encrypted search scheme supporting fault tolerance and verifiability in hybrid clouds [19]. This scheme utilized a dictionary-based index construction method to generate a fuzzy dictionary, enabling dynamic sorting and the verifiability of query results. Kurosawa et al. leveraged RSA accumulators to generate query tokens, facilitating the verification of query results while supporting dynamic record updates [20]. In 2015, Ameri et al. developed a general multi-level verifiable keyword search scheme using Bloom filters and a multi-level access control mechanism [21]. In 2017, Jiang et al. designed a verifiable encrypted search scheme supporting multi-label ranking, by utilizing a special inverted index structure along with binary vectors and MAC functions [22]. In 2020, Yang et al. proposed a verifiable semantic encrypted search scheme, designing a verification mechanism that uses intermediate data produced during the matching process to verify the correctness of queries [23]. In 2022, Li et al. proposed a verifiable ranked fuzzy multi-keyword search scheme using the homomorphic MAC technique and a random challenge technique to verify the correctness and completeness of returned results [24]. In 2023, Li et al. constructed a verifiable Boolean encrypted search scheme with a multiset hash function, achieving both forward and backward privacy [8]. In 2024, Zhang et al. designed a fine-grained encrypted query verification algorithm for cloud-assisted edge computing [25].

Blockchains have also been utilized in the context of verifiable encrypted search. In 2018, Cai et al. [26] proposed a fair encrypted search framework based on smart contracts, which verifies results by simulating the index and search process. In 2020, Tang et al. [27] shifted the responsibility of arbitration to a smart contract, eliminating the need for volunteers to ensure fairness. However, the index reconstructions and search simulations within the smart contract incur significant validation overhead. In the same year, Xixi Yan et al. proposed a verifiable attribute-based encrypted search scheme [14], which integrates blockchain technology to address the issue of incorrect query results returned by a semi-honest-but-curious cloud server. In 2021, Najafi et al. introduced a multi-label dynamic encrypted search and query result integrity verification method [9], to meet the demands of multi-key encrypted searching in practical applications. In 2023, PH Kumar et al. proposed the HMAC-Rijndael framework, which combines multiple homomorphic verifiable tags to create an identity-based dynamic system capable of cloud data integrity auditing and block hash merging [28]. In the same year, He et al. proposed a privacy-preserving method for verifiable fuzzy keyword searches based on the Ethereum blockchain in a cloud context, to overcome the aforementioned security concerns [29].

### 2.2. Secure Key-Value Datastores

Another closely related area of research is secure key-value datastores. In 2011, Popa et al. developed CryptDB, which provides encrypted query processing to protect key-value data confidentiality, using various encryption techniques to achieve multi-level data protection [30]. In 2017, Yuan et al. presented EncKV, a system that supports rich queries over encrypted key-value data while ensuring strong data confidentiality [31]. In 2018, Mishra et al. introduced Oblix, an efficient oblivious search index for secure data storage systems [32]. These works illustrate the evolving landscape of secure and private key-value storage systems. In 2020, Gu et al. proposed a locally differentially private key-value data collection framework that utilizes correlated perturbations to enhance utility [33]. In 2021, Li et al. introduced authenticated key-value stores with hardware enclaves [34]. In 2023, Wu et al. introduced novel poisoning attacks on local differential privacy protocols for key-value data, both theoretically and empirically [35]. In 2024, Zhang et al. proposed an encrypted and compressed key-value store with pattern-analysis security and minimal overhead [36].

In 2019, Nathan et al. designed and implemented a secure blockchain key-value database, leveraging serializable snapshot isolation to guarantee that replicas across nodes remain consistent [16]. In 2022, Li et al. proposed a hierarchical searchable encryption scheme using blockchain-based indexing, suitable for applications with fine-grained access requirements [37]. In 2023, Chen et al. presented ChainKV, a semantics-aware key-value storage paradigm aimed at improving storage management performance for the Ethereum system [38].

However, these methods either offer limited expressiveness in validating query requests or suffer from heavy computational loads, making them unsuitable for verifying Boolean-encrypted key-value queries. Furthermore, many verifiable encrypted search schemes require users to maintain large auxiliary structures, leading to substantial local storage burdens and increased costs for verifying the correctness of Boolean query results. These limitations make such schemes impractical for mobile and IoT devices with limited resources, especially in scenarios that require real-time key-value data analysis—such as streaming data from IoT sensors.

## 3. Preliminaries

### 3.1. Blockchain Data Store

A blockchain data store is a distributed append-only dictionary data structure [14,15,16,17], which is tamper-resistant, such that the records, once inserted, cannot be deleted without compromising the integrity of the entire structure. Designing EKV-VBQ based on a blockchain data store means that our key-value data structure cannot be modified or deleted without any data owner’s permission. We instantiate our blockchain data store using BDS = (Setup, Get, Put):

- Setup$(1^{k})\to B$: This protocol creates a blockchain wallet that includes a public address for the B. The public address is used solely for funding the wallet.
- Put$\left(B,v\right)\to (B^{\prime },r\left(v\right))$: This protocol stores the value *v* in a transaction, and signs and sends it to the blockchain. The address r(v) of the value varies depending on the blockchain structure. For some, it is the transaction hash; for others, it is the transaction hash along with the block number in which the transaction was mined.
- Get(B,r(v))→v: This protocol communicates with one or more nodes to retrieve the transaction corresponding to the address r(*v*) and then retrieves the value *v* stored in that transaction.

We use Ethereum blockchains, leveraging its proof of stake (PoS) consensus mechanism, which provides the essential properties and functionality needed for our blockchain-based key-value data structure. In PoS, blocks are finalized very quickly—typically within seconds—and the transactions included in these blocks achieve immediate finality, preventing any forks or rollbacks. By integrating Ethereum’s PoS mechanism, EKV-VBQ achieves both security and performance, ensuring that data, once inserted, cannot be modified or deleted, while still providing efficient query and storage operations.

### 3.2. Bilinear Accumulator

An accumulator is a constant-time-authentication data structure that can verify the identity of an arbitrary number of values [39]. A bilinear accumulator aggregates all elements of a set by representing the set with a characteristic polynomial, and it serves as an effective tool for proving the membership of elements in the set [40]. A bilinear accumulator can construct a constant-time digest for any large dataset and provide proof for any element in the set, verifying its membership. We use a symmetric bilinear accumulator as a module to verify Boolean operations in EKV-VBQ. The construction is as follows:

Let G be a multiplicative cyclic group of prime order *p* with a generator *g*. Let G be another multiplicative cyclic group of the same order. Choose a bilinear pairing instance (p,G,G,e,g), where *p* is the order of the integer group, and the set ϵ in $Z_{p}$ contains *n* elements $\left(e_{1},e_{2},\cdots ,e_{n}\right)$. Let *s* be the private key of the accumulator, and let $\left(g,g^{s},\cdots ,g^{s^{q}}\right)$ be the public key of the accumulator, where *q* should be greater than the number of elements in the set ϵ. The accumulated value of the set ϵ in G is defined as

$$\mathrm{acc}\left(\epsilon \right)=g^{\prod_{e\in \epsilon }\left(s+e\right)}$$

where *s* is a random value in $Z_{p}^{*}$, and where $\prod_{e\in \epsilon }\left(s+e\right)$ is an *n*-th degree characteristic polynomial of *s*, i.e., $f\left(s\right)=\prod_{e\in \epsilon }\left(s+e\right)$. The proof for the element $e_{i}$ being in the set ϵ is

$$\Omega_{e_{i},\epsilon }=g^{\prod_{e\in \epsilon -e_{i}}\left(s+e\right)}$$

where $\prod_{e\in \epsilon -e_{i}}\left(s+e\right)$ is an (n−1)-th degree polynomial of *s*.

The verifier needs to determine if the element $e_{i}$ is in the set ϵ by verifying the equation $\left(e\left(\Omega_{e_{i},\epsilon },g^{s+e_{i}}\right)=e\left(\mathrm{acc}\left(\epsilon \right),g\right)\right)$. Additionally, the accumulator can be used to prove the subset relationship. For sets Σ and ϵ, the subset membership proof for Σ⊆ϵ is

$$\Omega_{\Sigma ,\epsilon }=g^{\prod_{e\in \epsilon -\Sigma }\left(s+e\right)}$$

The verifier can check whether Σ is a subset of ϵ using the equation $\left(e\left(\Omega_{\Sigma ,\epsilon },g^{\prod_{e\in \epsilon -\Sigma }\left(s+e\right)}\right)=e\left(\mathrm{acc}\left(\epsilon \right),g\right)\right)$. The verifier can compute acc(ϵ) using precomputed values $\left(g,g^{s},\cdots ,g^{s^{q}}\right)$ without knowing the private key *s*. The security of the bilinear pairing accumulator is based on the bilinear *q*-strong Diffie–Hellman assumption.

## 4. The Model of EKV-VBQ

### 4.1. Architecture

EKV-VBQ is designed to operate among a data owner O, a service provider (server) S, and a blockchain network B consisting of multiple distributed nodes.

The data owner O initializes the key-value data structure locally, then encrypts and divides it into linked lists, which are sent as transactions to the blockchain network to create a secure key-value data store I. Based on the transaction addresses and additional auxiliary verification elements, O generates a dictionary D, which is also encrypted locally and then sent to S. The incremental storage capability of the blockchain structure allows I to meet the dynamic update requirements, enabling O to add key-value records at any time while simultaneously updating the dictionary D.

Subsequently, O can generate a Boolean query token τ by encrypting the query labels $L_{q}=\left\{l_{1},\cdots ,l_{q}\right\}$ in the Boolean expression $f(L_{q})$, and it sends τ to S. Upon receiving τ, S communicates with B to retrieve the records in I according to τ and D, it computes the Boolean result $R=f(R_{1},\cdots ,R_{q})$, and it generates proofs π to verify the correctness of the Boolean operations. Upon receiving the results and related proofs (R,π), O verifies the correctness locally. If the verification passes, it confirms that S has correctly executed the Boolean query.

### 4.2. Syntax

The syntax of EKV-VBQ consists of four polynomial-time algorithms and protocols, i.e., EKV-VBQ = (Init, Append, Query, Verify), described as follows:

- Init($1^{k}$)→(K,pp,I,D): Initialization algorithm, executed by O. It inputs security parameter $1^{k}$, outputs secret key *K* and public parameter pp, a secure key-value structured datastore I, and an encrypted lookup dictionary D.
- Append(O(K,(l,v)),S(D),B(I))$\to (⊥;D^{\prime };I^{\prime })$: Append protocol, executed interactively by O, S, and B. O inputs secret key *K*, key-value record (l,v); S inputs encrypted lookup dictionary D, B inputs the secure key-value structured datastore I. This protocol outputs updated $D^{\prime }$ and $I^{\prime }$.
- Query($O\left(K,f\left(L_{q}\right)\right),S\left(D,pp\right),B\left(I\right)$)→((R,π);⊥;⊥): Query protocol, executed interactively by O, S, and B. O inputs secret key *K*, Boolean label expression $f(L_{q})$; S inputs encrypted lookup dictionary D and the public parameters pp; B inputs the secure key-value structured datastore I. This protocol outputs Boolean query result *R* and correctness proof π.
- Verify(K,R,π)→1/0: Verification algorithm, executed by O. It inputs secret key *K*, query result *R*, and proof π, and outputs 1 or 0, indicating acceptance or rejection.

### 4.3. Threat Model

In EKV-VBQ, we define the following threat assumptions:

1. Honest-but-curious adversary: We assume that the blockchain B is honest but curious regarding its accuracy, and that it shares all of its internal status with the general public. It attempts to obtain the plaintext of the key-value structured datastore I but does not possess the secret key to access the data.
2. Malicious adversary: Unlike a traditional cloud server, the server S in EKV-VBQ is considered a malicious adversary. It attempts to obtain the plaintext of the data and queries, but the lookup dictionary D stored on S and the query labels remain encrypted at all times. Furthermore, the server may forge Boolean query results for incentives, such as saving storage and computation resources.
3. Trusted party: In realistic scenarios, we assume that the data owner O is fully trusted throughout the entire process.

### 4.4. Security Definition

The security of EKV-VBQ consists of two aspects: (1) the key-value structured datastore I and the encrypted lookup dictionary D should not leak any information about the records to S and B; (2) the query results contain no omissions and fully contain all the records matching the queried labels, which means they have not been maliciously altered and have not been tampered with or forged; and (3) S should not forge, modify, or tamper with the Boolean operation process of the query results. Under the above security properties, EKV-VBQ should satisfy indistinguishability under chosen label attack (IND-CLA secure), Integrity, and Unforgeability.

#### 4.4.1. IND-CLA Secure

IND-CLA security ensures that the server S and the blockchain nodes in B cannot retrieve any information about the label from the encrypted key-value datastore and the query token, even if they can conduct a polynomial-time query protocol. Let A be an adversary, Sim be a simulator, and $L_{1}$, $L_{2}$, $L_{3}$ be leakage functions for the Init, Append, and Query phases, respectively. We define the following two experiments:

${\mathrm{Real}}_{A}^{\text{EKV-VBQ}}\left(1^{k}\right)$: An interactive experiment between the adversary A and the challenger C using the real scheme. In this experiment, the challenger C runs Init to generate the key *K* and public parameter pp. The adversary A outputs a record set *F* to the challenger C, who runs the Init to produce I and B and sends them to the adversary A. The adversary A can make polynomially adaptive queries $\left(L_{i},f\left(L_{i}\right)\right)$ to the challenger C. For each query, the challenger C generates a query token $\tau_{i}$ for the adversary A. Since A is adaptive, the result of each query can be used as input for the next query. After *q* queries, the adversary A outputs a bit *b* as the output of the experiment.


${\mathrm{Ideal}}_{A}^{\text{EKV-VBQ}}\left(1^{k}\right)$: An interactive experiment between the adversary A and the simulator Sim. Based on the leakage functions $L_{1}$ and $L_{2}$, the simulator Sim generates a simulated $\tilde{I}$ and $\tilde{D}$ and sends them to the adversary A. The adversary A can make polynomial-time adaptive queries $\left(L_{i},f\left(L_{i}\right)\right)$ to the simulator Sim. For each query, the simulator Sim can access the leakage function $L_{3}$ and return the corresponding simulated query token $\tilde{\tau_{i}}$. Finally, the adversary A returns a bit *b* as the output of the experiment.

**Definition** **1** (IND-CLA Secure). *EKV-VBQ satisfies IND-CLA security if and only if for all polynomial-time adversaries A, there exists a polynomial-time simulator Sim, such that*

$$\left|\mathrm{Pr}\left[{\mathrm{Real}}_{A}^{\mathrm{EKV-VBQ}}\left(1^{k}\right)\right]-\mathrm{Pr}\left[{\mathrm{Ideal}}_{\mathrm{A,Sim}}^{\mathrm{EKV-VBQ}}\left(1^{k}\right)\right]\right|\leq \mathrm{negl}\left(1^{k}\right)$$

*where $\mathrm{negl}(1^{k})$ is a negligible function.*

#### 4.4.2. Integrity

Integrity ensures that the query results have not been forged or tampered with. Specifically, the system guarantees that for any given Boolean label expression, the corresponding query results completely contain all records matching the queried labels, meaning that all results for each queried label must be included in the returned set.

**Definition** **2** (Integrity). *EKV-VBQ satisfies Integrity if and only if for all security parameters $1^{k}$ all (I,D) are generated by Init$\left(1^{k}\right)$, and for a polynomial number of Append(O(K,(l,v)),S(D),B(I)) operations, for any given Boolean label expression $f(L_{q})$, Query$(O\left(K,f\left(L_{q}\right)\right),S\left(D,pp\right),B$(I)) will always output a result R, such that there does not exist a $v_{i}\notin R$ where $v_{i}$’s label $l_{i}\in L_{q}$:*

$$\left|\mathrm{Pr}[v_{i}\notin R\wedge l_{i}\in L_{q}]\right|\leq \mathrm{negl}\left(1^{k}\right)$$

*where $\mathrm{negl}(1^{k})$ is a negligible function.*

#### 4.4.3. Unforgeability

Unforgeability means that the server S should not forge, modify, or tamper with the Boolean operation process of the query results. For all security parameters *k*, all (K,I,D) generated by Init, any sequence of Append protocol by polynomial pairs (l,v) to update (I,D), Query($O\left(K,f\left(L_{q}\right)\right),S\left(D,pp\right),B\left(I\right)$) always produces the correct result *R* for any Boolean label expression $f(L_{q})$, i.e., Verify(K,R,π) always outputs 1. Otherwise, if *R* is not the correct Boolean result of $f(L_{q})$ then Verify(K,R,π) will output 0. Let A be a stateful adversary; let C be a challenger; consider the ${\mathrm{Forge}}_{A}$($1^{k}$) game, where the adversary A outputs a simulated record set {(l,v)} to the challenger C, who runs Init and Append to produce I and the encrypted lookup dictionary D and sends them to the adversary A. The adversary A can make polynomially adaptive queries $\left(l_{i},f\left(l_{i}\right)\right)$ to the challenger C. After *q* queries, the challenger sends a Boolean expression $f(L_{q})$ to A, the adversary A outputs an incorrect result $R^{\prime }$, and a proof π for $R^{\prime }$. If the proof π passes the verification algorithm Verify(*K*, $R^{\prime }$, π) = 1 then the experiment outputs 1; otherwise, it outputs 0. The formal description of ${\mathrm{Forge}}_{A}^{EKV-VBQ}\left(1^{k}\right)$ is
$$\begin{array}{cc} \; & {\mathrm{Forge}}_{A}^{EKV-VBQ}\left(1^{k}\right):\\ \; & \left(K,pp,I,D\right)\leftarrow \mathrm{Init}\left(1^{k}\right)\\ \; & \delta \leftarrow A(1^{k})\\ \; & \left(I^{\prime },D^{\prime }\right)\leftarrow \mathrm{Append}\left(K,\delta ,I,D\right)\\ \; & \mathrm{for}\;1\leq i\leq \;q\\ \; & \;\left\{L_{i}\right\}\leftarrow A\left(I,D,\tau_{1},\ldots ,\tau_{i-1}\right)\;\\ \; & \;\;\;\;\;\;\tau_{i}\leftarrow \mathrm{Query}\left(K,f\left(L_{i}\right)\right),\;\\ \; & \left(f\left(L_{q}\right),R^{\prime },\pi \right)\leftarrow A\left(I,D,\tau_{1},\ldots ,\tau_{q}\right)\\ \; & \mathrm{output}\;b\leftarrow \mathrm{Verify}(K,R^{\prime },\pi ) \end{array}$$

**Definition** **3** (Unforgeability). *EKV-VBQ satisfies Unforgeability if and only if for all polynomial-time adversaries A we have*

$$\mathrm{Pr}\left[{\mathrm{Forge}}_{A,C}^{EKV-VBQ}\left(1^{k}\right)=1\right]\leq \mathrm{negl}\left(1^{k}\right)$$

*where $\mathrm{negl}(1^{k})$ is a negligible function.*

## 5. Design Ideas

### 5.1. Encrypted Key-Value Structured Datastore

**Figure 1:**
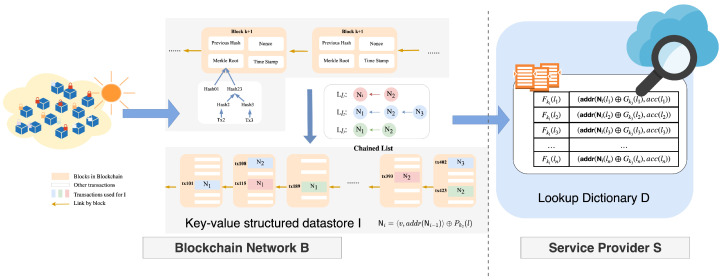
An illustration of encrypted key-value structured datastore.

The encrypted key-value structured datastore for EKV-VBQ consists of an incremental chained structure I stored in B and an encrypted lookup dictionary D stored in S. The structure is illustrated in Figure 1:

To initial I, O generates *n* virtual linked lists $\left(L_{l_{1}},\cdots ,L_{l_{n}}\right)$ organized by labels $L\;=\;(l_{1},\cdots ,l_{n})$. Each list consists of nodes with a unique record of the same label, which is stored in a transaction of the blockchain network. The structure of a node $N_{i}$ in list $L_{l}$ is defined as $\langle v,r\left(N_{i-1}\right)\rangle$, where *v* is the unique record and $r(N_{i-1})$ is the transaction address of the previous node in $L_{l}$. Then, it is XOR-encrypted, using $P_{k_{3}}\left(l\right)$ as $N_{i}=\langle v,r\left(N_{i-1}\right)\rangle ⊕P_{k_{3}}\left(l\right)$ to protect the privacy of the real key-value records against the blockchain network. Additionally, to allow S to perform a Boolean query in I, O initials a key-value dictionary D that contains all label information as the keys. For each label *l*, the corresponding value in D is the transaction address of the head node in the linked list $L_{l}$, i.e., $r(N_{\#N_{L_{l}}})$ and the accumulator value acc(l). Using pseudo-random function *F*, O encrypts the label *l* into $F_{k_{1}}\left(l\right)$ and encrypts the corresponding value as $\langle r\left(N_{1}\right)⊕G_{k_{3}}\left(l\right),acc\left(l\right)\rangle$: $D\left[f_{k_{1}}\left(l\right)\right]=\langle r\left(N_{1}\right)⊕G_{k_{3}}\left(l\right),acc\left(l\right)\rangle$.

To append new key-value record (l,v), O should retrieve the transaction address r(N) of the last head node N in the chain $L_{l}$. O communicates with B to update the chain $L_{l}$ by appending a new head node $N^{\prime }$ corresponding to the new record *v*: $N^{\prime }=\langle v,r\left(N\right)\rangle ⊕P_{k_{3}}\left(l\right)$ and obtains the transaction address $r(N^{\prime })$. Additionally, O computes the new accumulator value $acc^{\prime }\left(l\right)$ based on the previous one acc(l) and the new record *v*, encrypts it as $\langle r\left(N^{\prime }\right)⊕G_{k_{3}}\left(l\right),acc^{\prime }\left(l\right)\rangle$, and interacts with S update $D\left[F_{k_{1}}\left(l\right)\right]$ as $\langle r\left(N^{\prime }\right)⊕G_{k_{3}}\left(l\right),acc^{\prime }\left(l\right)\rangle$.

### 5.2. Boolean Queries and Verifications

O interacts with S to execute the query process for the result of the Boolean expression $f(L_{q})$ on labels $L_{q}=\left(l_{1},l_{2},\ldots ,l_{q}\right)$. For each label $l_{i}\in L_{q}$, O generates the query token $t_{l_{i}}=\left(F_{k_{1}}\left(l_{i}\right),P_{k_{2}}\left(l_{i}\right),G_{k_{3}}\left(l_{i}\right)\right)$; sending $\tau =({\left\{t_{l_{i}}\right\}}_{l_{i}\in f\left(L_{q}\right)},f\left(L_{q}\right))$ to S.

S retrieves $\left(\theta_{1},\theta_{2}\right)$ in $D[F_{k_{1}}\left(l_{i}\right)]$, uses $f_{2}k_{3}\left(l_{i}\right)$ to XOR $\theta_{1}$, and obtains the transaction address of the head node of $L_{l_{i}}$ in I. S then communicates with B to retrieve the transaction and XORs the transaction data, using $P_{k_{2}}\left(l_{i}\right)$ to obtain the record identifiers and the addresses of the next nodes in $L_{l_{i}}$. After extracting the information of all nodes in the $L_{l_{i}}$, S then gathers results $\left(R_{1},\cdots ,R_{m}\right)$. It subsequently performs the Boolean operations (intersection, union, or difference) based on $f(L_{q})$. Depending on the operation type, the correctness of the Boolean operation must satisfy the following properties:

As an intersection $R_{I}=R_{l_{1}}\cap R_{l_{2}}\cap \ldots \cap R_{l_{q}}$, it must be a subset of each set $R_{l_{i}}$, i.e., $R_{I}\subseteq R_{l_{i}}$; and it must contain all common labels of the sets $\left(R_{l_{1}},R_{l_{2}},\ldots ,R_{l_{q}}\right)$. The proof for the Boolean operation is shown in Section 6, so it is not elaborated here.

## 6. The Detailed Construction

### 6.1. Initialization

First of all, O initializes a dictionary D with N entries, where $N>2^{k}$; it generates a key-value structured datastore I by calling $\mathrm{Setup}\left(1^{k}\right)$, which is instantiated by the Ethereum web3 API package [41] with MetaMask [42] creating a local wallet. For each label l∈L:

1. Construct a linked list $L_{l}$ with a head node $N_{1}$; the structure of $N_{1}$ is defined as $N_{1}=\langle ⊥,0\rangle ⊕P_{k_{2}}\left(l\right)$.
2. Call signTransaction() and sendRawTransaction() to instantiate Put$\left(I,N_{i}\right)$, which encapsulates the node $N_{1}$ as a transaction into the blockchain network, and obtain its transaction address $r(N_{1})$.
3. Select a randomness *r* and calculate the initial accumulated value of *l*: $acc\left(l\right)=g^{s+r}$.
4. Set $D\left[f_{k_{1}}\left(l\right)\right]=\langle r\left(N_{1}\right)⊕G_{k_{3}}\left(l\right),acc\left(l\right)\rangle$.

Moreover, O randomly selects three *k* bit strings $k_{1},k_{2},k_{3}$ and aggregates the key *K* as $K=(k_{1},k_{2},k_{3},s)$; it selects bilinear pairing parameters (p,G,G,e,g) and $s\in Z_{p}^{*}$ as a random secret trapdoor; and it computes $\left(g,g^{s},g^{s^{2}},\cdots ,g^{s^{q}}\right)$ as the public parameter pp. Then, O sends D to S.

### 6.2. Append

To append a new key-value record (l,v), O communicates with the service provider S and the blockchain network B to update $I^{\prime }$ and $D^{\prime }$. First, O computes $\tau_{a}=F_{k_{1}}\left(l\right)$ and sends $\tau_{a}$ to S. S retrieves $\left(\theta_{1},\theta_{2}\right)\leftarrow D\left[t_{a}\right]$ and sends $\left(\theta_{1},\theta_{2}\right)$ back to O. O then generates $N_{i}=\langle v,\theta_{1}\rangle ⊕P_{k_{2}}\left(l\right)$, calls signTransaction() and sendRawTransaction() to instantiate $\mathrm{Put}(B,N_{i})$, encapsulates the node $N_{i}$ as a transaction value into the blockchain network, obtains the transaction address $r(N_{i})$, computes $\theta_{1}^{\prime }=r\left(N_{i}\right)⊕G_{k_{3}}\left(l\right)$, generates the accumulated value of *l*: $acc\left(l\right)=\theta_{2}^{\prime }=\theta_{2}^{s+v}$, then sends $\left(\theta_{1}^{\prime },\theta_{2}^{\prime }\right)$ back to S. S receives $\left(\theta_{1}^{\prime },\theta_{2}^{\prime }\right)$ from O and sets $D\left[t_{a}\right]=\langle {\theta_{1}}^{\prime },\theta_{2}^{\prime }\rangle$.

### 6.3. Query

To query the Boolean expression $f\left(L_{q}\right)=\left\{I,U,D\right\}$ on the label set $L_{q}=\left\{l_{1},l_{2},\ldots ,l_{q}\right\}$, for each $l_{i}\in L_{q}$, O generates the query token $t_{l_{i}}=\{F_{k_{1}}\left(l_{i}\right),G_{k_{2}}\left(l_{i}\right),$$P_{k_{3}}\left(l_{i}\right)\}$, and sends the query token $\tau =\{{\left\{t_{l_{i}}\right\}}_{l_{i}\in L_{q}},f\left(L_{q}\right)\}$ to S, where *f* is the Boolean expression.

For each ${\left\{t_{l_{i}}\right\}}_{l_{i}\in L_{q}}$, S initializes an empty set $R_{l_{i}}$ and retrieves the label, as follows: $\left(\theta_{l_{i},1},\theta_{l_{i},2}\right)=D\left[t_{l_{i},1}\right]$; it computes the transaction address in I: $r=\theta_{l_{i},1}⊕t_{l_{i},2}$, it calls *getTransaction()* to instantiate tx←Get(r), it inputs the transaction address r, it retrieves the associated transaction content, it reads the data field to extract node information *tx* from I, it computes $a_{1}\left|\right|a_{2}\leftarrow tx⊕t_{l_{i},3}$, it sets $a_{2}$ as new address r, and it adds $a_{1}$ in the result $R_{l_{i}}$. S repeats the above steps until r=0.

S generates the accumulator set $T={\left\{\theta_{l_{i},2}\right\}}_{l_{i}\in L_{q}}$ and computes the Boolean result $R_{f(L_{q})}$ based on the results $R_{l_{1}},R_{l_{2}},\ldots ,R_{l_{q}}$. S then generates the correctness proof of the Boolean result $R_{f(L_{q})}$, which is determined by Boolean operation types ($a_{1}$: interaction; $a_{2}$: union; $a_{3}$: difference):

- $a_{1}$: S computes the intersection $R_{I}=R_{l_{1}}\cap R_{l_{2}}\cap \ldots \cap R_{l_{q}}$, supposing $R_{I}=\left(v_{1},\cdots ,v_{m}\right)$; for each set $R_{l_{i}}$, the public parameter $\left(g,g^{s},\ldots ,g^{s^{q}}\right)$ is used to compute the polynomial $P_{i}=\prod_{v\in R_{l_{i}}-R_{I}}\left(s+v\right)$; the subset inclusion proof $I_{R_{I},R_{l_{i}}}$ is computed for the set $R_{I}$ with respect to $R_{l_{i}}$: $I_{R_{I},R_{l_{i}}}=g^{P_{i}}$; the subset proof $I={\left\{I_{i}\right\}}_{1\leq i\leq q}={\left\{I_{R_{I},R_{l_{i}}}\right\}}_{1\leq i\leq q}$ is computed; given $\left\{P_{1},\ldots P_{m}\right\}$, the extended Euclidean algorithm is used to find the polynomial $\left\{q_{1},\ldots ,q_{m}\right\}$, such that it satisfies the completeness condition $q_{1}P_{1}+q_{2}P_{2}+\cdots +q_{m}P_{m}=1$; the completeness proof is set as C=$\left\{g^{P_{1}},\ldots ,g^{P_{m}}\right\}$. S integrates the intersection proof $\pi_{R_{I}}=\left\{T,I,C\right\}$.
- $a_{2}$: S computes the union $R_{U}=R_{l_{1}}\cup R_{l_{2}}\cup \ldots \cup R_{l_{q}}$, supposing $R_{U}=\left(v_{1},\cdots ,v_{m}\right)$ for each set $R_{l_{i}}$, and each record $v_{j}$ in O executes the following steps: use the public parameter $\left(g,g^{s},\ldots ,g^{s^{q}}\right)$ to compute the polynomial $F_{i,j}=\prod_{v\in R_{l_{i}}-v_{j}}\left(s+v\right)$; compute $M_{i,j}=g^{F_{i,j}}$; compute the member proofs and superset proofs $M={\left\{M_{i,j}\right\}}_{1\leq i\leq q,1\leq j\leq m}$, $S={\left\{S_{i}\right\}}_{1\leq i\leq q}={\left\{g^{P_{i}}\right\}}_{1\leq i\leq q}$. S integrates the union proofs $\pi_{R_{U}}=\left\{T,M,S\right\}$.
- $a_{3}$: Given the difference query token $\tau =(D\left(l_{i},l_{j}\right),\tau_{i},\tau_{j})$, S computes the difference $R_{D}=R_{l_{i}}-R_{l_{j}}$. Supposing $R_{D}=\left\{v_{1},\ldots ,v_{m}\right\}$, the server then computes the proofs $\pi_{R_{D}}=\left\{M,S\right\}$ as follows: For each record *v* in $R_{D}$, use the public parameter $\left(g,g^{s},\ldots ,g^{s^{q}}\right)$ to compute the polynomial $v_{1}=\prod_{v\in R_{D}-v_{k}}\left(v+s\right)$, $v_{2,1}=\prod_{v\in R_{li}-v_{k}}\left(v+s\right)$, $v_{2,2}=\prod_{v\in R_{lj}-v_{k}}\left(v+s\right)$. S computes $M=(M_{1},M_{2},M_{3})$, where $M_{1}=g^{v_{1}}$, $M_{2}=g^{v_{2,1}},M_{3}=g^{v_{2,2}}$; compute the intersection $R_{l_{i}\wedge l_{j}}=R_{l_{i}}⋂R_{l_{j}}$; compute $\mathrm{acc}\left(R_{l_{i}\wedge l_{j}}\right)=g^{\prod_{v\in {R_{I}}_{l_{i}\wedge l_{j}}}\left(v+s\right)}$; for the sets $R_{D}$ and $R_{l_{i}\wedge l_{j}}$, compute $P_{1}=\prod_{v\in R_{l_{i}}-R_{D}}\left(v+s\right)$, $P_{2}=\prod_{v\in R_{l_{i}}-{R_{I}}_{l_{i}\wedge l_{j}}}\left(v+s\right)$, $P_{3}=\prod_{v\in R_{D}}\left(v+s\right)$, and $P_{4}=\prod_{v\in {R_{I}}_{l_{i}\wedge l_{j}}}\left(v+s\right)$. S computes the superset proofs $S=\left(S_{1},S_{2},S_{3},S_{4}\right)=(g^{P_{1}},g^{P_{2}},$$g^{P_{3}},g^{P_{4}})$ and integrates the difference proofs $\pi_{R_{D}}=\left\{T,M,S\right\}$.

S then sends the query result set $R_{f(L_{q})}$ and the corresponding proofs π to the data owner O.

### 6.4. Verify

O can verify the Boolean query results. It outputs 1 or 0, indicating acceptance or rejection. The main steps are as follows:

- $a_{1}$: O verifies the subset condition by checking the equation $e\left(g^{\prod_{k=1}^{m}\left(s+v_{k}\right)},I_{i}\right)=e\left(T_{i},g\right)$, where the elements $\left(v_{1},\ldots ,v_{m}\right)$ are elements in $R_{I}$, and where $I_{i}=g^{P_{i}}$ are elements in the subset proof S; $a_{1}$: O verifies the completeness condition, using the following equation: $\prod_{i=1}^{m}e\left(S_{i},C_{i}\right)=e\left(g,g\right),$ where $S_{i}=g^{P_{i}}$ are elements in the subset proof S, and where $C_{i}=g^{q_{i}}$ are the corresponding elements in the completeness proof C. If the above verification process passes, output 1 indicates successful verification; otherwise, output 0 indicates verification failure.
- $a_{2}$: for each record $v_{j}\in R_{U}$, 1≤j≤m, O verifies the membership condition through the following equation: $e\left(M_{i,j},g^{v_{j}}g^{s}\right)=e\left(T_{i},g\right),$ where $M_{i,j}=g^{F_{i,j}}$ is an element in M. For each set $R_{l_{i}},1\leq i\leq n$, we verify the superset condition through the following equation: $e\left(S_{i},T_{i}\right)=e\left(g^{\prod_{k=1}^{d}\left(s+v_{k}\right)},g\right),$ where $S_{i}$ is an element in the superset proof S. If the membership condition for any record in $R_{U}$ does not hold for any set $R_{l_{i}}$ then verification fails. If the above verification process passes, output 1 indicates successful verification; otherwise, output 0 indicates verification failure.
- $a_{3}$: For each $v_{k}\in R_{l_{i}},1\leq k\leq m$, O verifies the membership condition through the following equations: $e\left(M_{1},g^{v_{k}}g^{s}\right)=e\left(acc\left(R_{D}\right),g\right)$ $e\left(M_{2},g^{v_{k}}g^{s}\right)=e\left(T_{i},g\right)$ $e\left(M_{3},g^{v_{k}}g^{s}\right)=e\left(T_{i},g\right),$ where $\left(M_{1},M_{2},M_{3}\right)=(g^{v_{1}},g^{v_{2,1}},$$g^{v_{2,2}})$ are elements in the membership proof M. O verifies the superset condition through the following equations: $e\left(S_{1},S_{3}\right)=e\left(g^{\prod_{k=1}^{d}\left(s+v_{k}\right)},g\right)$ $e\left(S_{2},S_{4}\right)=e\left(g^{\prod_{k=1}^{d}\left(s+v_{k}\right)},g\right),$ where $\left(S_{1},S_{2},S_{3},S_{4}\right)=\left(g^{P_{1}},g^{P_{2}},g^{P_{3}},g^{P_{4}}\right)$ are elements in the superset proof S. If any of the above three membership condition equations do not hold, verification fails. If the above verification process passes, output 1 indicates successful verification; otherwise, output 0 indicates verification failure.

## 7. Security Analysis

### 7.1. IND-CLA Secure

Theorem 1 shows that, given leakage functions $L_{1}$, $L_{2}$, and $L_{3}$—which describe the information leaked to the server during the initialization, append, and query operations, respectively—EKV-VBQ satisfies IND-CLA secure in the random oracle model, which is defined in Definition 1.

**Theorem** **1.** *If F, G, and P are pseudo-random functions then EKV-VBQ is secure against adaptive chosen label attacks in the random oracle model, i.e., it satisfies IND-CLA secure, which is defined in Definition 1.*

**Proof.**  In ${\mathrm{Ideal}}_{\mathrm{A,Sim}}^{\mathrm{EKV-VBQ}}\left(1^{k}\right)$, based on the information leaked in $L_{1}$ and $L_{2}$, the simulator Sim can determine the size of D and I and the label domain L in ${\mathrm{Real}}_{A}^{\text{EKV-VBQ}}\left(1^{k}\right)$. Then, it can use random values to construct the simulated $D^{\prime }$ and $I^{\prime }$ as the simulated data store.

1. Since the real D is encrypted using XOR and pseudo-random functions *F* and *G* in ${\mathrm{Real}}_{A}^{\text{EKV-VBQ}}\left(1^{k}\right)$, and since the simulated one is filled with random bits of the same length, if the pseudo-randomness of functions *F*, *G* holds then for all PPT adversaries A it is impossible to distinguish between the real D and the simulated $D^{\prime }$ in polynomial time.
2. Since the key-value stores I in ${\mathrm{Real}}_{A}^{\text{EKV-VBQ}}\left(1^{k}\right)$ consist of shuffled nodes that are a bunch of transactions in B, and since each node contains one label/value pair and is encrypted using pseudo-random functions *P*, and since the simulator can generate simulated nodes with random bits of the same length, it is the case that if the pseudo-randomness of functions *P* holds then for all PPT adversaries A it is impossible to distinguish between the real I and the simulated $I^{\prime }$ in polynomial time.

To respond to the adversary A’s queries, the simulator Sim needs to use $L_{3}$ to construct simulated query tokens that are indistinguishable from real tokens. Specifically, the leakage function $L_{3}$ reveals the identifiers id(v) of all labels l∈L, as well as the association between each label identifier *l* and the record identifiers containing the label *l*. Because $\tilde{\tau }=\left({\tilde{t}}_{1},{\tilde{t}}_{2},\ldots ,{\tilde{t}}_{q}\right)$, ${\tilde{t}}_{i}=(\gamma \left((l_{i})\right),T\left[\gamma \left((l_{i})\right)\right]⊕\mathrm{iT}\left(\left(l_{i}\right)\right),$$k_{\left(l_{i}\right)})$, and $t_{i}=\left(F_{k_{1}}\left(l_{i}\right),G_{k_{2}}\left(l_{i}\right),P_{k_{3}}\left(l_{i}\right)\right)$, based on the pseudo-randomness of functions *F*, *G*, and *P* for all PPT adversaries A it is impossible to distinguish the query token τ from the simulated query token $\tilde{\tau }$.

In conclusion, for all PPT adversaries A the outputs of the experiments ${\mathrm{Real}}_{A}\left(k\right)$ and ${\mathrm{Ideal}}_{\mathrm{A,S}}\left(k\right)$ are indistinguishable, except with negligible probability $\mathrm{negl}(1^{k})$. Therefore, EKV-VBQ satisfies IND-CLA secure in the random oracle model.      □

### 7.2. Integrity

**Theorem** **2.** *If the blockchain consensus mechanism is maintained then EKV-VBQ satisfies the Integrity property defined in Definition 2.*

**Proof.**  EKV-VBQ ensures integrity by leveraging Ethereum’s consensus mechanism. The smart contracts execute predefined logic for search operations and store results publicly and immutably as contract states on the blockchain. Each node in the network can independently detect any tampering with these results, making any alterations easily identifiable. Unlike traditional schemes that assume the server performs searches honestly, EKV-VBQ removes the need for this assumption. The decentralized nature of the blockchain, combined with the consensus mechanism, ensures that miners within the Ethereum network verify the correctness of each search operation. This distributed verification guarantees that query results have not been altered or forged.

Thus, according to the definition of Integrity (Definition 2), EKV-VBQ ensures that for any Boolean label expression $f(L_{q})$ all records matching the labels in $L_{q}$ are included in the result set *R* and there is no $v_{i}\notin R$ such that $l_{i}\in L_{q}$. Therefore, EKV-VBQ satisfies the Integrity property as defined.      □

### 7.3. Unforgeability

**Theorem** **3.** *If the bilinear q-SDH assumption holds then EKV-VBQ satisfies Unforgeability as defined in Definition 3: that is, for any probabilistic polynomial-time adversary A it is impossible to generate valid proof for a forged Boolean query result.*

**Proof.**  If there exists a probabilistic polynomial-time adversary A such that the output of the experiment ${\mathrm{Forge}}_{A}\left(1^{k}\right)$ is 1 then there exists a simulator Sim capable of breaking the bilinear *q*-SDH assumption. The proof mainly focuses on the Unforgeability in the intersection Boolean query scenario, where union and difference are similar, and it will not be repeated here. If an adversary can forge a valid proof then a simulator Sim can break the collision resistance of the hash function $H_{3}$ and the *q*-strong Diffie–Hellman assumption, at least. Thus, for any probabilistic polynomial-time adversary A the probability of winning the experiment ${{\mathrm{Forge}}_{A}}^{EKV-VBQ}$($1^{k}$) is a negligible function $\mathrm{negl}(1^{k})$ concerning the security parameter $1^{k}$. Therefore, EKV-VBQ possesses Unforgeability.      □

## 8. Theoretical Analysis

### 8.1. Complexities

We provide a comprehensive theoretical analysis and evaluation of the complexity of EKV-VBQ in Table 1. Let *n* represent the domain size of the label set, *M* the upper bound on the number of key-value records for each label, *q* the number of labels included in a Boolean query request, and *m* the number of records in the Boolean query results. The cost analysis of the scheme is summarized in Table 1. The storage cost for the data owner O is O(1) for storing the key locally, and for the service provider S it is O(n) to store the dictionary D, which consists of *n* entries with two *k*-bit strings. The storage cost for I in the blockchain network is O(Mn). The computation cost for O to construct the query token is linear in the number of query labels *q*, i.e., O(q). For S, the cost to perform the query is O(qn). The cost to perform intersection operations and generate proofs is $O(n\log^{2}n\log\log n)$, while the cost to perform union and difference operations and generate proofs is O(nlogn). The computation cost for O to verify the Boolean results is O(q)+O(m). The communication cost during a query is primarily determined by the number of query labels and the number of records in the results. When O queries a set of *q* labels, it interacts with S in only one round. The communication complexity for sending the token is O(q), and for S to return the query results it is O(m). In summary, the total data size is O(q+m).

### 8.2. Comparisions

We compare our scheme with the related schemes [8,10,16,24,25,37,38,43] in Table 2. Due to the significant differences among the existing schemes in application scenarios, secure models, evaluation indicators, and other factors, we focus on comparing characteristics and security. In these related schemes, most studies assume the presence of malicious attackers in their threat models, highlighting a strong emphasis on security. Refs. [8,9,43] provide search and Boolean verification mechanisms, which implement comprehensive verification functionalities. The performance of update efficiency varies, with [10,25,37] lacking a clear update mechanism. The types of blockchain employed are diverse, including permissioned and public blockchains, while consensus mechanisms vary, with [16,37] using proof of work (PoW) and [10] opting for proof of stake (PoS); [25,37] demonstrate support for distributed trust.

Compared with other schemes, EKV-VBQ offers significant advantages by supporting Boolean encrypted queries to enhance result integrity, and it operates securely under a malicious threat model. Additionally, EKV-VBQ allows for updates, ensuring the database remains dynamic, and it leverages Ethereum for decentralized trust. The use of proof of work (PoS) further strengthens data integrity and security, making EKV-VBQ a robust solution for privacy-preserving data aggregation and querying, and making it more suitable for a real-life thin clients MSNs deployment scenario.

## 9. Experimental Analysis

We implemented and evaluated EKV-VBQ, to analyze its efficiency. The server side was deployed on AWS p3.2xlarge instances in Seattle USA) instances equipped with NVIDIA Tesla V100 GPUs in California USA utilizing the PyTorch framework, version 1.10.0 csprng library [44] for cryptographic operations. The client (data owner) experimental environment was set up on a Linux Ubuntu 18.04.2 64-bit machine equipped with an Intel Core i7-2600 quad-core processor (3.4 GHz) and 8 GB of RAM. Multiple hosts were deployed to simulate a client interacting with the server.

We used the Ethereum simulation platform—Ropsten Testnet [45]—to simulate the blockchain network B, a public blockchain based on proof of work that supports smart contracts, and we utilized the Ethereum web3 API package [41] to construct the simulated secure key-value structured datastore I. We generated the experimental data synthetically. We created our key-value database with 100,000 randomly generated key/value pairs. To ensure high security, we employed a 256-bit security parameter and implemented the pseudorandom functions *F*, *G*, and *P*, using the OpenSSL 3.4.0 [46] algorithm. Additionally, we utilized LevelDB [47] to store the encrypted lookup dictionary D. Each experiment was run multiple times (ranging from 50 to 100 iterations), and the average results were reported, to analyze EKV-VBQ’s performance.

### 9.1. Communication Overhead

**Figure 2:**
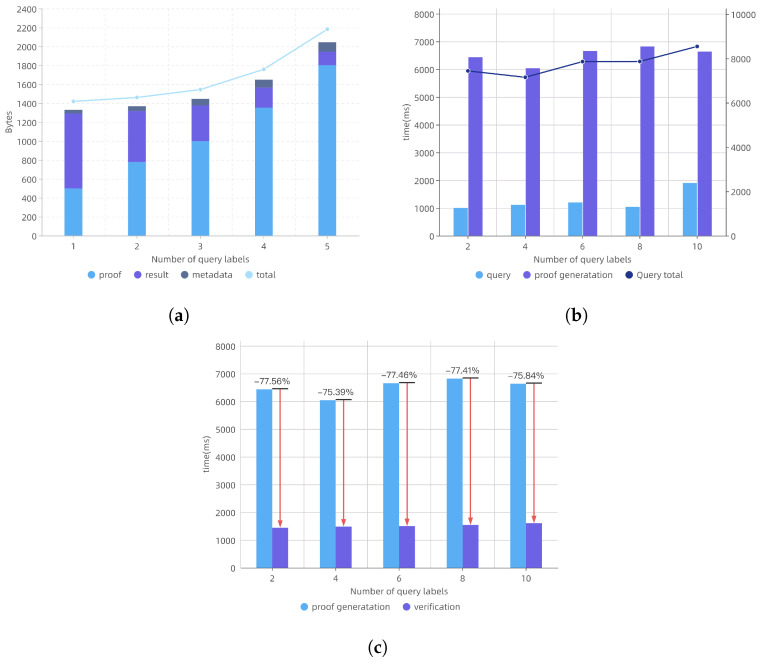
Cost evaluations: (**a**) communication overhead for different query labels; (**b**) query time, proof generation time, and total query time across different query labels; (**c**) proof generation and verification time across different query labels.

We analyzed the communication overhead between the server and clients during a query, which primarily consisted of the data volume of the query results and the additional proof metadata. Figure 2a illustrates the relationship between communication overhead and the number of labels in the Boolean expression. As the number of query labels increased (2, 4, 6, 8, 10), the size of the query results in the communication overhead decreased from 792.05 to 141.01 bytes. This decrease occurred because the query results that matched multiple labels in the Boolean expression were reduced, leading to a corresponding decrease in the transmitted data volume. Conversely, the proof data volume increased from 499 to 1803 bytes, as more labels required additional proof data to ensure the correctness and completeness of the query results, accounting for 37.5% to 88.1% of the total data volume. Therefore, the additional proof data was the primary factor influencing the communication overhead, which aligned with the theoretical analysis.

### 9.2. Computation Overhead

**Figure 3:**
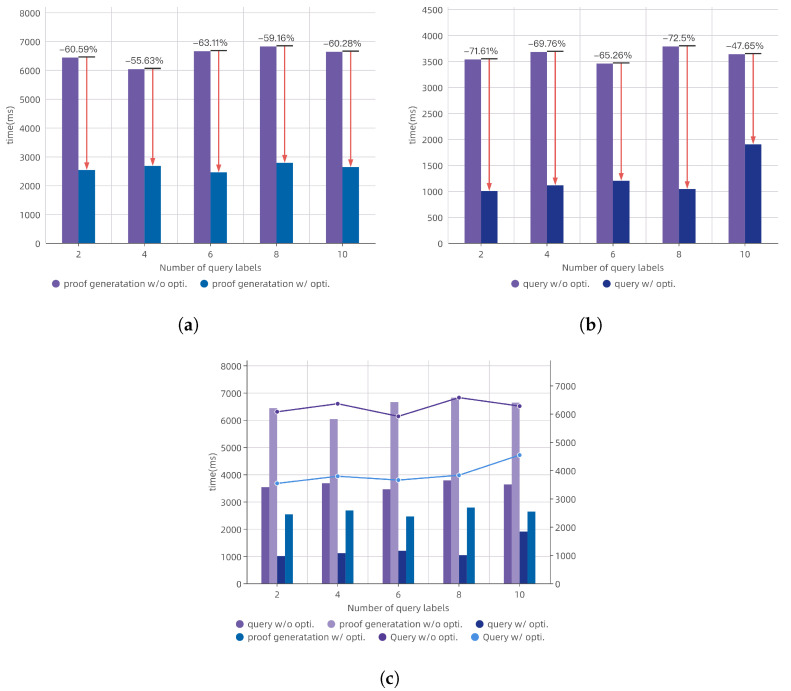
Optimization evaluations: (**a**) proof generation times with and without optimization. (**b**) query times with and without optimization.(**c**) overall query times with and without optimization.

The computation overhead comprised the query overhead and verification overhead, as illustrated in Figure 3b,c. Figure 2b demonstrates that the time overhead for querying the blockchain was approximately linear with respect to the number of labels. The proof generation process took longer than the query process, due to the more complex exponentiation and bilinear pairing operations. Despite the variation in the number of labels, the time overhead remained relatively stable, which was consistent with the theoretical analysis.

The verification overhead primarily consisted of the server-side proof generation overhead and client-side local verification overhead. For the client-side local verification overhead, we primarily measured the time required for the client to complete the verification operation with different numbers of labels. As shown in Figure 2c, the verification overhead was independent of the number of labels, remaining between 1.4 and 1.6 s. The most time-consuming operation during the verification was the bilinear pairing process. Compared to the server-side proof generation time, the client’s computation overhead was between 75.39% and 77.56% of that. Therefore, EKV-VBQ imposed relatively low computation overhead on the client, with efficient and fast verification operations, significantly reducing the client’s computational burden.

### 9.3. Optimization

The computation overhead was significantly influenced by the chosen curve parameters and pairing algorithms. Multi-threaded computation and pre-computation could substantially reduce computation time during the proof generation phase. Since the Queries required repeated access to the blockchain network, these retrieval operations were highly parallelizable and could be accelerated using GPUs. We replicated the lookup dictionary D within each warp (32 threads) of each streaming multiprocessor (SM), and we placed the replicated D in the shared memory of each SM on the GPU, which helped mitigate stalls caused by accessing D. This optimization reduced the required time by 53%. As shown in Figure 3, this optimization significantly decreased the time needed for both the query processing and the proof generation. The improvements ranged from approximately 47% to 72% for query processing and from 55% to 63% for proof generation.

Overall, the evaluation results underscore EKV-VBQ’s scalability and efficiency, establishing it as a robust solution for secure key-value query applications.

## 10. Discussion

In this section, we discuss the extensions and future research directions or potential applications of EKV-VBQ:

Advanced blockchain integration: Future research could explore incorporating EKV-VBQ into newer blockchain platforms, such as delegated proof of stake (DPoS) systems. These platforms offer lower energy consumption and faster consensus, which could reduce the computational and time overhead of EKV-VBQ operations. Additionally, such integration would enhance scalability, allowing for greater data throughput while maintaining verifiability. Researchers could also investigate the use of sharding or sidechains to further improve system efficiency. This would make EKV-VBQ more suitable for large-scale applications, such as enterprise or governmental use cases.

Mobile and IoT applications: EKV-VBQ’s lightweight verification protocols and optimization for GPU acceleration make it ideal for resource-constrained environments. Future work could focus on further reducing the computational load and energy consumption of the protocol, to make it more practical for mobile devices and internet of things (IoT) applications. This could involve hardware-specific optimizations, such as integration with energy-efficient processors or edge computing frameworks. These improvements would allow secure and verifiable queries in real-time IoT networks, such as smart cities or remote healthcare monitoring systems. This direction could open up new possibilities for large-scale sensor networks with privacy concerns.

Support for more complex queries: While EKV-VBQ currently supports Boolean queries, future research could extend its functionality to include more complex query types. This could involve adding support for multi-conditional range queries, fuzzy searches, or keyword proximity searches, all while preserving the integrity and privacy of the data. Researchers might focus on optimizing the underlying cryptographic structures, to maintain efficiency even with these additional query types. Such extensions would broaden the scope of EKV-VBQ, making it applicable in fields like natural language processing or geospatial data analysis. Ensuring that these complex queries remain verifiable is key to the scheme’s long-term viability.

Multi-party computation (MPC): Future research could explore integrating EKV-VBQ with multi-party computation (MPC) frameworks, enabling multiple data owners to collaboratively perform secure and verifiable queries without revealing their private inputs. This would be particularly useful in scenarios involving multiple stakeholders, such as federated learning or secure data sharing in healthcare systems. The combination of MPC and EKV-VBQ could enhance both privacy and trust, ensuring that all parties could verify query results independently. Researchers could focus on optimizing communication and computational overhead for MPC use cases, making it scalable for real-world deployment. This development would extend EKV-VBQ’s utility in collaborative environments requiring strong privacy guarantees.

Healthcare and finance applications: EKV-VBQ could be adapted to handle privacy-sensitive data in sectors like healthcare and finance, where the confidentiality of queries and data integrity are critical. For instance, verifiable Boolean queries could be applied to encrypted medical records, ensuring that sensitive patient data remained private while allowing for secure access by authorized parties. In the financial sector, EKV-VBQ could support secure auditing, fraud detection, and compliance checks on encrypted datasets. Researchers could focus on fine-tuning the system for regulatory compliance, such as GDPR or HIPAA. This would make EKV-VBQ a powerful tool for privacy-preserving analytics in highly regulated industries.

## 11. Conclusions

In this paper, we presented EKV-VBQ, a novel scheme designed to ensure verifiable Boolean queries in encrypted key-value datastores. EKV-VBQ leverages homomorphic encryption, bilinear accumulators, and blockchain technology to support verifiable Boolean query operations while preserving both data integrity and privacy. Our comprehensive security analysis demonstrated that EKV-VBQ resists adaptive chosen label attacks and is unforgeable under the bilinear *q*-strong Diffie–Hellman assumption. Our experimental evaluations confirmed that EKV-VBQ efficiently reduces the computational burden on the client side, making it well suited for resource-constrained environments. Through the optimization of verification algorithms and GPU acceleration, EKV-VBQ strikes a balance between security, efficiency, and practicality, providing a robust foundation for secure data outsourcing and query verification in real-world applications, including mobile and IoT environments, where computational resources are limited.

Future enhancements in verifiable computation techniques will further improve EKV-VBQ’s verification performance. Additionally, the scheme’s flexible design enables its potential adaptation to more complex query types, such as fuzzy or multi-conditional range queries, expanding its applicability to diverse domains, such as healthcare, finance, and large-scale sensor networks. Ultimately, EKV-VBQ sets a new standard for secure, efficient, and verifiable data management in privacy-sensitive environments.

## Figures and Tables

**Table 1:** Complexity analysis.

	Stor	${\mathrm{Comp}}_{A}$	${\mathrm{Comm}}_{A}$	${\mathrm{Comp}}_{Q}$	${\mathrm{Comp}}_{P}$	${\mathrm{Comm}}_{Q}$	${\mathrm{Comp}}_{V}$
User	O(1)	O(1)	O(1)	O(q)	-	O(q+m)	O(q+m)
Server	O(n)	O(1)	O(1)	O(qn)	O($n\log^{2}n\log\log n$)	O(q+m)	-

Stor: storage complexity; $Comp_{A}$: Append computation complexity; $Comm_{A}$: Append communication complexity; $Comp_{Q}$: Query computation complexity; $Comm_{Q}$: Query communication complexity; $Comp_{V}$: Verify computation complexity. *n*: number of records; *q*: number of query labels; *m*: number of query results; *M*: the upper bound of the number of records for each label.

**Table 2:** Properties comparison.

	Boolean Query	Encrypted Search	Boolean Verification	Threat Model	Update	Blockchain	Consensus Mechanism	Distributed Trust
[16]	×	✓	×	Malicious	✓	Permissioned BC	PoW	×
[37]	×	✓	×	Honest but Curious	×	Public BC	PoW	✓
[38]	×	×	×	×	✓	Ethereum	PoS	×
[24]	×	✓	×	Malicious	✓	×	×	×
[8]	✓	✓	✓	Malicious	×	×	×	×
[25]	×	✓	×	Malicious	×	✓	×	×
[9]	✓	✓	✓	Malicious	✓	×	×	×
[10]	×	×	×	Malicious	✓	Ethereum	PoS	×
[43]	✓	✓	✓	×	×	Ethereum	PoW	×
EKV-VBQ	✓	✓	✓	Malicious	✓	Ethereum	PoS	×

## Data Availability

Data are contained within the article.
